# On the Use of Embedded Fiber Optic Sensors for Measuring Early-Age Strains in Concrete

**DOI:** 10.3390/s21124171

**Published:** 2021-06-17

**Authors:** K.K. Santos Silva, F.A.N. Silva, T. Mahfoud, A. Khelidj, A. Brientin, A.C. Azevedo, J.M.P.Q. Delgado, A.G. Barbosa de Lima

**Affiliations:** 1 Civil Engineering Department, Pernambuco Catholic University, Recife 50050-900, Brazil; klayne.dos-santos-silva@2020.icam.fr (K.K.S.S.); fernando.nogueira@unicap.br (F.A.N.S.); 2 Research Institute in Civil Engineering and Mechanics, Centrale Nantes, CEDEX 3, 44321 Nantes, France; mahfoud.tahlaiti@icam.fr (T.M.); abdelhafid.khelidj@univ-nantes.fr (A.K.); antoine.brientin@icam.fr (A.B.); 3 CONSTRUCT-LFC, Civil Engineering Department, Faculty of Engineering, University of Porto, 4200-465 Porto, Portugal; antonio.costaazevedo@fe.up.pt; 4 Mechanical Engineering Department, Federal University of Campina Grande, Campina Grande 58429-900, Brazil; antonio.gilson@ufcg.edu.br

**Keywords:** embedded fiber optic sensors, fiber sensors, sensing techniques for concrete structures

## Abstract

Detailed information about concrete behavior in real structures is an important issue in controlling its performance during its service life, and the use of embedded sensors to obtain desired information such as temperature, onset of the cracking process and evolution of strains, has gained the attention of the building concrete industry. Data obtained using this technology can provide valuable information for decision making about the need for corrective interventions that can ensure the integrity and safety of concrete structures for long period of time. This paper presents a review of the current state-of-the-art of embedded fiber optic sensors used to assess concrete information of a wide range of aspects, comprising: existing alternative technologies, characteristics and advantages, practical applications and future developments. Complementarily, the work presents preliminary results of the use of fiber optic sensors to automatically and continuously perform expansion readings of AAR in concrete elements that facilitate both the storage—with elimination of the usual interruptions for manual readings—and the availability of continuous results of expansion data that are not possible to obtain with usual AAR tests code reading recommendations.

## 1. Introduction

Concrete is one of the main structural materials, widely used worldwide in several types of work and construction projects. Durability and performance to withstand mechanical loads are some reasons for its widespread use. As a heterogeneous material, made with constituents from different origins, concrete structures may exhibit pathologies during their useful life, due to both the applied loads and its interaction with the environment. Over time, significant scientific comprehension of the nature of this material has been developed, including the perception that certain problems may cause changes in its micro- and macro-structure with the possibility of generating an early deterioration process, and even the failure of the material [1,2,3]. The occurrence of such problems can result from an improper selection of types of aggregates and cement for making concrete mixtures, or it can arise from a delayed formation of some hydration products. Regarding the development of its compressive strength throughout time, some concretes may show pathologies due to environmental action, unforeseen stresses and external loads or due to changes in their micro-structure that can lead to a cracking process that reduces its durability and performance [4].

Structural health monitoring (SHM), with a focus on concrete structures, has emerged along with the need to increase the safety of structures and to provide guidelines for efficient and cost-effective maintenance plans. Currently, for the SHM approach, several point sensors are used, but due to the complexity of concrete structures, it is often necessary to use a large number of sensors. Fiber optic sensors (FOS) present great advantages in terms of multiplexing capabilities, chemical stability, resistance to electromagnetic field, compactness, etc. These advantages make FOS a safe technology for a large range of applications, namely, industrial, medical and structural health monitoring [5,6,7,8]. Fiber optic technology has been increasingly used to monitor concrete structures, and it can be useful in achieving integrated detection with a high degree of sensitivity, durability and stability [9,10,11].

To evaluate the behavior of concrete structures under normal loading conditions and during their use, it is essential to use safe monitoring strategies that allow material quality indicators to be obtained, in order to take decisions on possible intervention processes before the damage process increases. Over time, new technologies have been investigated, with the use of fiber optic sensors being one of the most promising [12,13,14,15,16,17].

The use of embedded FOS for reading expansions due to alkali-aggregate reaction (AAR) in concrete elements is a fairly new line of scientific investigation, because those readings are typically associated with the manual operations of removing the specimen from the environment in which it was placed with the subsequent several manual works that introduce undesirable errors in the measurements. The work presents preliminary results regarding the use of FOS to automatically and continuously perform expansion readings of AAR in concrete elements that facilitate both the storage—with elimination of the usual interruptions for manual readings—and the availability of continuous results of expansion data, which are not possible to obtain with usual AAR tests code reading recommendations.

## 2. Overview of Health Monitoring of Concrete Structures Using FOS

The occurrence of cracks in concrete directly affects the concrete’s service life and durability. Structural health monitoring (SHM) is becoming increasingly necessary for concrete structures as a complement to the visual inspections usually performed. Monitoring concrete structures is extremely important to identify problems early and to adopt the most appropriate recovery measure. In recent years, several SHM techniques have been developed for concrete structures using fiber optic sensors, piezoelectric materials and radioactive materials (X-rays and γ-rays) [18,19,20,21].

The use of fiber optic sensors is shown to be increasingly promising because the sensors exhibit a higher sensitivity, durability, and stability, when compared to traditional sensors, and also are a smaller size. In addition, the sensors can withstand high temperatures and environmental aggressions, which make the FOS ideal for SHM of large concrete structures [22,23,24,25].

Fan et al. [26] developed a real-time, in situ corrosion monitoring method using distributed helix pattern fiber optic sensors applied on a steel surface. The aim was to investigate the concrete deterioration under steel corrosion. A telecommunication-grade optical fiber consisted of a fused silica core and cladding and an acrylic primary and secondary coating, and a polymeric buffer layer was used as both the transmission line and a distributed sensor. Reinforced concrete beams made with three different concrete mixtures were prepared and instrumented with the distributed fiber optic sensors installed on the reinforcement surface. Corrosion was accelerated by submerging the beams in a NaCl solution and three concrete cover thickness and three water/cement ratio were investigated. The results reported indicate that the investigated approach of using a helix pattern of fiber optical sensors was proven to be feasible for in situ and real time monitoring of steel corrosion. The fiber optical sensor used was capable of capturing strains arising from corrosion phenomena of steel bars, during the 94 h of the tests. No report of degradation of the sensor material was reported, even in such an aggressive environment, but it should be highlighted that duration of the tests was not very long; about 4 days.

Liao et al. [27] developed a system to monitor a concrete pavement slab subjected to external temperature fluctuations. Three types of sensors were used, including traditional thermocouples as surface temperature sensors, a temperature and strain embedded fiber optic and an optical fiber inclinometer. Fiber optic sensors were protected with a primary coating. Strain and temperature were measured during heating and cooling of concrete and the values obtained show that these values were not evenly distributed on each slab layer studied. The authors reported that the strain exhibited a positive relationship with the temperature change, regardless of the gravity effect. The system was shown to be effective in detecting the thermal curling of the slab and the temperature/strains distribution profiles were well captured and the fiber optic sensors demonstrated an ability to continuously monitor temperature and strain. Although the limits of the layout used by the authors does not allow a visualization of the nonlinear profile distribution of temperature and strain, slight adaptations may allow this behavior to be captured.

Waeystens et al. [28] used fiber optic sensors to measure mechanical strain in an 8 m simply supported post-tensioned concrete beam. The strategy developed by the authors aimed to update physical parameters associated with damages in concrete (usually related to changes in the mechanical properties of the material) to minimize the gap between the numerical strains obtained from finite element analysis and the strains from sensor outputs. The authors used the optical fibers output coupled with physical models with a continuous reduction of Young’s modulus of the material. They divided the beam’s damaged area into several subdomains where the respective Young’s modulus was updated using the best fitting among finite element simulations and sensor outputs. The proposed model succeeded in capturing the damage in the concrete element investigated.

Scott et al. [29] developed a cost-effective and easy-to-use technique for encapsulating resin fiber optic sensors, using a 3D printing technique sensor system suitable for use with concrete structures for both surface mounting and embedment situations. Tests performed by the authors demonstrated the potential of the packaged sensors for strain measurement in concrete structures, but further development is still needed to assess the quality of their applicability.

Tan and Bao [30] developed a method to measure crack widths with distributed fiber optic sensors installed in special test specimens designed by the author to create cracks with different opening sizes. The authors used three types of telecommunication-grade single-mode optical fibers as distributed fiber optic sensors, including a bare fiber and two coated fibers. Fiber optic sensors were used to read the evolution of crack widths in a two aluminum plates and two U-shaped cross section steel bars. The crack widths were calculated based on the strain distribution measured from the distributed sensors. The authors reported that the results from the distributed sensor and the extensometer agreed well with each other and sensor outputs can be used to calculate crack widths. A method to quantify crack widths was proposed considering that the strain peak can be correlated to the crack width. The results obtained indicated that the strategy used was efficient for measuring crack widths at a micro- and macro-scale as well as being precise enough to detect the onset of cracking.

Fiber optic sensors offer a number of advantages over conventional electrical measuring gauges and transducers to calculate strains and displacements, to determine the onset of the cracking process and the crack opening widths, and to identify localized damage and other important information, in order to monitor concrete structures performance.

Surface or embedded FOS have also been used in many newly constructed civil structures—bridges, buildings, dams, pavements, pile cap blocks—providing technical information about static and dynamic behavior, temperature, wind or water pressure, and structural health, allowing for continuous real-time monitoring of structural behavior [31,32]. Even amongst non-specialists in using sensors, there is a consensus that SHM is a promising strategy, and there is a high level of confidence in them when it comes to monitoring concrete structures.

The main characteristics that make FOS potential candidates for SHM of concrete structures are the following: (1) FOS consist of an electrically nonconductive material; (2) FOS can be safely used in an explosive atmosphere; (3) FOS are not subject to electromagnetic interference; (4) FOS can be chemically inert; (5) FOS have a very wide range of operating temperatures; and (6) the sensors can be multiplexing.

On the other hand, practical application in a field environment demands the observation of some special technical issues such as fragility, ways of installation (surface or embedded), deterioration and connectors; factors that should be carefully investigated before the selection of a specific FOS package to use. These issues are very important if one intends to use FOS in in situ and real-time monitoring of expansions in concrete elements due to internal swelling reactions (ISR).

### 2.1. Lab Tests with FOS in Concrete

The work developed by Yehia et al. [33] used optical fibers to measure strains due to physical changes, such as heat of hydration, as well as strains due to cyclical and torsional loads. Concrete cylinders of 15 × 30 cm^2^ and beams of 175 cm in length and with a cross section of 35 × 55 cm^2^ were used. Fiber optic sensors were inserted in the cylinders (one in each) and in the beams (two in each). The FOS sensors used were the embedded fiber optics (e-FOS), temperature-compensated and strain gauge for cylinder and beam testing, respectively. The sensors are based on Fabry–Perot interferometry.

To identify temperature variations during the hydration of the concrete, thermocouples were installed in the middle and on the surface of the beams, in order to detect temperatures between 40 °C and 100 °C. All sensors were connected to a data acquisition system right after molding to record strains due to the heat of hydration and also the shrinkage of the concrete. The same procedure was used for both beams and cylindrical specimens. Monitoring was performed continuously for 7 days.

The results, provided by the sensors, for each test performed on the concrete samples, as a function of time, showed an instantaneous response to any changes in loading as well as temperature changes, which indicates the sensitivity and the ability of the FOS to capture responses of the concrete specimens due to loading or during hydration.

Another research that used FOS to read concrete strains was developed by Sienko et al. [29]. In this work, fiber optic sensors were used to investigate strains and cracks in a reinforced concrete element during a load test. Strains were measured, analyzing the cracks that occurred during the load application. The crack widths were calculated based on the fiber deformation data obtained. FOS-type single-mode telecommunication optical fiber SM 9/125 in a tight jacket (outer diameter of 0.9 mm) were used. The studied concrete element was 77 × 110 × 1050 mm^3^ with a 16 mm diameter steel bar inside, located in the middle of the cross section. The optical fibers were placed along the length of the element in six different places. After processing the raw data, the authors defined measurement bases in post-processing. The length of the bases taken was 10 mm and the distance between successive bases was 5 mm. This means that the sensor bases overlapped by half their lengths.

The experimental results showed strains of the fibers individually, according to each measurement base defined by the authors [34], before the concrete cracked. It was possible to observe that the fibers located close to the surface of the element (where surface shrinks occur), presented greater variations compared to the others. Tensile loading was eccentrically applied, so that cracks were initially formed in the upper part of the element and, due to the progressive redistribution effect, followed to the lower surface. It was observed that all critical cracks appeared in the element up to a load of 10 kN. It should be reported that significant changes in the signal presented by the fibers represent internal changes (strains) in the structural element. These strains were positive or negative, depending on the degree of crack development along the shortest edge of the cross section. This work [34] confirmed the usefulness of fiber optic technology to identify very small cracks (about 0.01 mm). This conclusion has an important practical significance, as it refers to cracks that are difficult to identify using other methods. In addition, the research also confirmed the utility of optical fiber strain measurements technology to analyze larger cracks, visible to the naked eye.

In another work, Horch et al. [32] went beyond the existing laboratory studies and used sensors in the foundation element of a tall building. For the research, a system called “Backscattered Optical Reflectometry” (OBR) was used. The configuration of the sensor system used had a spatial resolution of 5 mm, a temporal resolution of 50 Hz and a maximum detection of 20 m, for a structural element with 32 m in length. Altogether, 12 fibers were used, coupled to the reinforcement: eight for strain measurements and four for temperature. Regarding the eight sensors, four had cables with a diameter of 3.2 mm and four had cables with a diameter of 7.2 mm, to allow the comparison of the influence of the cable sizes to be made.

Reference measurements were made when the structure was 18 weeks old. Follow-up further readings were made when the structure was 26 weeks and 34 weeks old.

Temperature measurements were made using sensitive fibers able to read strain values close to 10 µm/m. This was performed in order to allow the levels of thermal expansion of the concrete to be compensated.

The temperature results obtained exhibited large ranges of oscillation (noise). This fact was reported by the authors as a non-consistent behavior that was assigned to some alteration coming from the cables or the machine. On the other hand, the temperature values oscillated around a constant average value throughout the foundation slab. The aim of the work developed by Horch et al. [32] was to demonstrate the use of fiber optic sensors distributed in a real building, and some logistics to install and read data were reported. Problems found did not allow a rational comprehension regarding the temperature and the influence of the different dimensions of the cables.

### 2.2. Embedded FOS to Health Monitoring of Concrete

Yuan et al. [35] developed a pre-embedded fiber optic concrete bar sensor (PECB) to use for real-time monitoring of concrete structures. The authors tested the PECB in cement-paste, cement-sand mixture material and a concrete pre-embedded concrete bar sensor, using a white light-fiber optic interferometer sensing system. The PECB developed consisted of a fiber optic sensor gauge, and input and output connection systems with a typical bar shape. In addition, the authors proposed a simple mechanical model for evaluating the effects of thermal induced expansion strains on the PECB in cement-based materials. The results obtained indicated that the thermal expansion behavior of the PECB reproduced the expected theoretical prediction domain well.

Li et al. [36] developed a theoretical model for measuring strains in concrete elements subjected to biaxial compression using embedded fiber optic sensors. The authors adopted some initial assumptions, as follows: (i) fiber cores and concrete matrix behave elastically; (ii) fiber optic coatings follow an elastoplastic behavior and (iii) both the conc rete matrix-coating surfaces and core-fiber surfaces are assumed to be undamaged. The authors used the sensing system developed by [32] and uniaxial and biaxial compression tests in cement mortar prisms. Single mode strain sensors based on interferometer theory were used and the calibration of the model was accomplished using special factors developed. Stress-strain curves in uniaxial compression obtained using strains readings with extensometers showed to be quite similar to those obtained using fiber optic sensors. For the biaxial compression tests, stress-strain curves in the x and z axes showed a good overall correlation, but some strain underestimations measured with fiber optic sensors were observed, when compared with those obtained using extensometers.

Kesavan et al. [37] investigated the performance of techniques of embedding fiber optic sensors in concrete. The authors tested four different methods: a pair of flat acrylic sheets, a liquid epoxy, a pair of cast epoxy sheets and a rod assembly. The authors reported that the best performance was obtained using the epoxy sheet encapsulation and embedment using rod assembly. Embeddable-type fiber optic sensors (both types of embedment techniques) were installed in cylinder specimens of concrete (150 mm in diameter and 300 mm in height) during the casting operations. Concrete cylinders were tested under compressive loading. Uniaxial test results showed that the embedded fiber optic sensors’ behavior was quite similar to that obtained using strain gauges, up to the elastic limit.

The authors also reported that the embedded optical sensors kept working with no visible damage or degradation, even after attaining a strain value close to 2000 με. For flexural behavior, the authors reported a quite efficient performance, although some strain underestimations had been observed, when compared with the results from usual strain gauge measurements. Despite this fact, the 7% of variation that was reported is usually accepted for SHM of concrete structures. The same behavior was observed in fatigue, in high stress levels and in low cycle loadings.

Bassil et al. [38] studied the strain transfer mechanism between a host material and an optical fiber. The authors developed an analytical model to deal with imperfect bonding between layers. The proposed model was verified using wedge-splitting tests in concrete specimens instrumented with surface and embedded fiber optical sensors, simultaneously with LVDTs that were used as a reference for displacement readings. The wedge-splitting test is a new method used to perform stable fracture mechanics tests to determine fracture energy values for concrete and concrete-like materials. According to the authors, this test was used because it creates a single crack vertical propagation and the strain in the host material is primarily located during the micro-cracking phase in the fracture zone and disappears later, during the macro-cracking phase. The strains in concrete can therefore be neglected, and it becomes more direct to measure the crack-induced strains. Several fiber optic cables were tested and the results showed that most of them exhibited similar behaviors, characterized by a linear relationship between peak strains and crack openings, until a given threshold. Beyond this limit, the sensors no longer behaved linearly. A good agreement among measured and calculated strain distribution was observed, with differences never exceeding 20% of the measured strains. Regarding the measurements of cracking openings, some optical fibers showed small relative errors (2%), and embedded ones exhibited larger errors (10%), for cracking openings varying in the range 200–1500 με.

Bao et al. [39] investigated the feasibility of using telecommunication single-mode optical fiber to measure strains and cracks in concrete pavements. The authors performed tests in full-scale concrete panels under truck and three-point loads to quantify the performance of sensors embedded in concrete. Sensors were specially protected with precast mortar to avoid damages during concrete casting operations. The authors concluded that, depending on their layout, the distributed sensors can provide one- or two-dimensional strain fields in pavements. The widths of both micro- and macro-cracks were reported to be linearly related to the peak strain, a fact already highlighted in previous work. Cracks were adequately detected at the location of the peak in the strain distribution profile, compatible with the physical measurement of the crack location in the concrete panel. Overall results obtained demonstrated that the distributed embedded fiber optic sensors in concrete are a good strategy to perform structural health monitoring of concrete structures. Nevertheless, the usual fragility of optical fiber demands appropriate procedures to be used their installation.

### 2.3. FOS to Assess Internal Strains in Concrete Affected by ISR

Fiber optic sensors can be used to measure strains in concrete elements. It is well-known that internal swelling reactions cause expansions due to the stresses generated by the formation of hydration products (expansive gel, ettringite crystals, etc.). The use of FOS specifically for the analysis of structures that present these types of pathology (AAR or DEF) is an open field to scientific research and qualified information about this subject is still very scarce.

One of the works that used FOS to assess internal expansion in concrete was developed by Dunant and Scrivener [40]. The authors used reactive and non-reactive coarse aggregates to produce concrete specimens that were tested under creep loading. The concrete was cured at a temperature of 20 °C for 28 days, and after this curing period the concrete specimens were placed in a room with a controlled temperature of 38 °C. It is important to highlight that the expansive reactions (ASR) were not accelerated. Concrete samples were finally placed in creep frames and subjected to 0, 5, 10 and 15 MPa stress levels to perform the creep tests.

In this work [40], three standard cylindrical concrete specimens (160 mm in diameter and 335 mm in height) were loaded in creep. The applied stress remained constant throughout the tests and the strains due to creep and induced ASR expansions were fully monitored. Embedded strain and temperature sensors were used and the specimens were kept undisturbed to compensate for strains due to temperature fluctuation. Results reported by the authors showed a cracking process starting at a stress level close to 5 MPa. This process often happened on the coarse aggregate surface. The authors also reported a strong relationship between ASR expansion and the direction of the micro-cracking process propagation, around coarse aggregates and the cement paste.

Rocha et al. [41] developed research studies using FOS in lab tests and real scale concrete structures. In the lab tests, 12 concrete samples made with potentially reactive aggregates were prepared to analyze their behavior related to AAR. Before molding the concrete, 12 distributed optical fiber strain sensors (DTSS) were inserted inside the molds. To perform external measurements, metal pins were fixed on the lateral surfaces of each sample. Each group of pins was fixed at least 50 cm apart. After 7 days, the specimens were demolding and placed in a tank with a 1N NaOH solution to accelerate the reaction, with a constant controlled temperature of 80 °C. External measurements were made daily for 30 days (the duration of the tests) and inner strain readings, performed with the fiber optic system installed, were made every 30 min. The authors reported cable degradation due to the aggressiveness of the NaOH solution at high temperature. Although these factors have not been considered in the field installations, they were relevant in the analysis of the lab tests because they affect the connection condition between the sensors and the surrounding expansion mortar.

The results of the tight buffer data were not reliable after 10 days of measurement, while the cable, which was a more protected element, showed consistent results up to the 15th day of measurements. After these periods of time in each case, the information obtained was reported as not relevant by the authors. However, the authors also reported that the expansion detected by the system, in the time interval in which it worked, results obtained were consistent with the measurements of the external pins. The values obtained were smaller than the external pin readings, as it was expected, due to the gradient of deformation from the outer surface to the center of the sample. However, an important observation that should be highlighted was the FOS were capable of detecting the evolution of AAR expansions.

Rocha et al. [41] also reported the monitoring of the Peti Dam, built between 1941 and 1945. This dam is located in the state of Minas Gerais-Brazil. Unlike the studies previously presented in this paper, in the Peti Dam the installation of optical fiber sensors was carried out after its construction. In this dam, structural abnormalities have been observed due to AAR since 1972. Eight preexisting vertical wells, of different depths, were instrumented with fiber optic sensors distributed along the dam in depths varying from 3 to 30 m. One limitation reported by the authors was possibility of installing DTSS and FBG sensors in only in one direction. The column regions and the central section were instrumented, with a large number of sensors on the left column, where more pronounced degradations were reported. The results showed that the Peti Dam was still exhibiting signals of a slow and continuous expansion due to AAR, even more than 70 years after its construction. This work showed the importance of optical fiber sensors for evaluation of expansions due to internal expansion reactions.

Finally, although no studies have been found using fiber optic sensors only for evaluation and monitoring of expansion by DEF, it is important to consider that although the origin of AAR and DEF are different, the effects on concrete structures in a macroscopic view are quite similar, i.e., both cause expansion in concrete elements and often occur simultaneously. From the studies presented, it is possible to state that regardless of the internal reactions existing in the structure, the expansions can be adequately detected using FOS.

## 3. Measurement of Early-Age Concrete Temperature and Strain Using Embedded Sensor

The heat generated by cement hydration reactions usually imposes thermal strains in concrete elements that may lead to an early cracking process, which decreases its durability. The durability of concrete depends on the material quality and the exposure conditions, which, in turn, depends on the temperature, on the physical-chemical aggressiveness of the surrounding environment and, also, depends on the mechanical loading.

To evaluate the feasibility of using embedded sensors to assess temperatures and strains in concrete elements in early ages, mainly those arising from internal swelling reaction due to AAR, an experimental procedure in concrete specimens was developed. To create a favorable environment for the development of internal swelling reactions in the concrete, in addition to the effects of the hydration heat, the strategy prescribed in [42] was used. The use of embedded FOS for measurement is a fairly new line of scientific investigation, as expansions due to AAR involve a complex process of removing the specimen from the environment in which it was placed with the subsequent performance of various manual actions that can introduce errors in the readings performed. In this situation, the use of embedded FOS introduces important advantages.

In this very first phase of the research, three concrete prisms with dimensions of 75 × 75 × 285 mm^3^ were prepared, two of them were kept in the aggressive environment as prescribed in [42], and the last one was maintained in air conditions, to be used as the reference prism. To produce the concrete, Portland cement CEM I 52.5 and coarse aggregates with a high potential alkali-reactivity were used. To accelerate the internal expansion reactions, an alkali content of 1.25% sodium oxide (NaOH) by cement mass was added to the mixing water and the two prisms were placed inside a tank with an alkaline solution equivalent to the internal environment to prevent leaching, according to RILEM recommendation [42]. A thermocirculator was used to gradually increase the temperature inside the tank until its stabilization, at around 70 °C, which was then kept constant during the tests.

Fiber optics and temperature sensors were embedded in the concrete specimens during concrete casting. It is important to highlight that early-age strain measurements in concrete using fiber optic sensors are highly influenced by the time the sensor is placed in contact with the material and by the dependence on the emitting sensor signals during the hardening of the concrete, mainly the temperature level. Some discussions regarding these issues were made in previous sections of this paper, for the type of fiber optic sensor used in the research.

### 3.1. Equipment and System of Data Acquisition

Fiber Bragg grating sensors inscribed in optical fiber were used to obtain strain measurements inside concrete samples. The acquisition of the Bragg wavelengths was made with an HBM FS22 DI sensor, referred to later as Braggmeter or FS22. This device relies on a broadband laser source (1500 nm to 1600 nm) and continuous swept laser scanning to obtain real-time measurements of the Bragg wavelengths, with an acquisition rate ranging from 50 Hz to 1000 Hz. Up to four optical fibers, each one with several embedded FBG sensors, can be monitored simultaneously. A custom data acquisition program was developed to enable the acquisition and storage of Bragg wavelengths measured by the FS22 over the whole experiment. This was necessary because the native program that came with the device had some internal settings that did not meet the test demands. In particular, the minimum acquisition rate of 50 Hz would generate a large quantity of measurement data over several weeks, with only a fraction of it really being needed. In order to facilitate data analysis, the custom program was designed to obtain 10 measurements of the Bragg wavelengths over a few seconds, and to then select the median value, which would be used during data analysis. Once this was carried out for each FBG, the program would wait 10 min before repeating the process. The advantages of this method are twofold: it limits the amount of data collected, and at the same time limits the impact of a possible disturbance, thanks to the repetition of measurements over a few seconds. Moreover, because collected data are timestamped, this means that in case of an unfortunate event such as a power loss, it is possible to know precisely how much time has passed without measurement.

### 3.2. Characteristics of Fiber Sensor Used

The FBG sensors used were inscribed in silica core-cladding fibers with a polyimide coating by a research partner, with a phase mask technique and a 240 nm UV laser. Bragg wavelengths range between 1530 and 1550 nm, reflectivity of the FBG is around 30% to 40%, with a full width at half maximum (FWHM) of reflected wavelengths around 0.15 nm. Just before concrete sample casting, a temperature sensitivity characterization was performed to ascertain precisely the response of the four fibers with five FBG on each. The reference temperature (ambient air) was accurately measured with a PT100 temperature probe, whose accuracy was previously verified using a calibration furnace. The temperature sensitivity characterization was performed in two ways, either by putting the stress-free fiber in cold water with temperature monitored by a PT100 temperature probe, or in a furnace with temperature also being monitored with a PT100. The wavelength variation observed by the Braggmeter and the temperature variation detected by the PT100 ensured that the sensitivity of the FBG sensors was the same as that reported in the literature: about 10 pm/°C. These measurements have also revealed the very high sensitivity of FBG sensors to spurious disturbances. Table 1 shows the FBG sensors’ properties used for experimental data analysis.

### 3.3. Measurements on Fiber-Reinforced Concrete Specimens

To measure the evolution of strains and stresses during the setting phase of concrete specimens, an instrumentation based on fiber Bragg grating sensors was developed. The choice of such a type of sensor is explained by their low intrusivity (the sensors are limited to the size of the optical fiber, i.e., a few tens of micrometers in diameter), as well as their sensitivity to the physical parameters whose evolution is to be measured.

Each of the three concrete prism specimens described in Section 3 was instrumented by an optical fiber with FBG sensor inscribed in it, placed at mid-height in the center of the specimen. In order to be able to dissociate the effects of temperature and strain evolution on the variation of the Bragg wavelength, PT100 temperature sensors were used to assess the temperature at the core of the concrete samples; the temperature probe was also placed at mid-height, away from the FBG sensors. A reference PT100 was also used to monitor ambient air temperature in the room.

## 4. Laboratory Test Results and Discussion

According to the response of an FBG sensors, the total strain is given by Equation (1):

(1) $$\varepsilon_{Total}=\frac{\Delta L}{L}=\left(\frac{\Delta \lambda_{B}}{\lambda_{B}}-K_{T}\times \Delta T\right)\times \frac{1}{K_{\varepsilon }}$$

With *K*_*T*_ and *K*_*ε*_ being fiber-dependent constants of 7.8 × 10^−6^/°C and 0.78 × 10^−6^/(μm/m), respectively. However, in view of the significant temperature variations expected in concrete during the setting phase, the effects of thermal expansion cannot be neglected, which leads to Equation (2).

(2) $$\varepsilon_{Total}=\alpha_{concrete}\times \Delta T+\varepsilon_{shrinkage}=>\varepsilon_{shrinkage}=\varepsilon_{Total}-\alpha_{concrete}\times \Delta T$$

**Figure 1:**
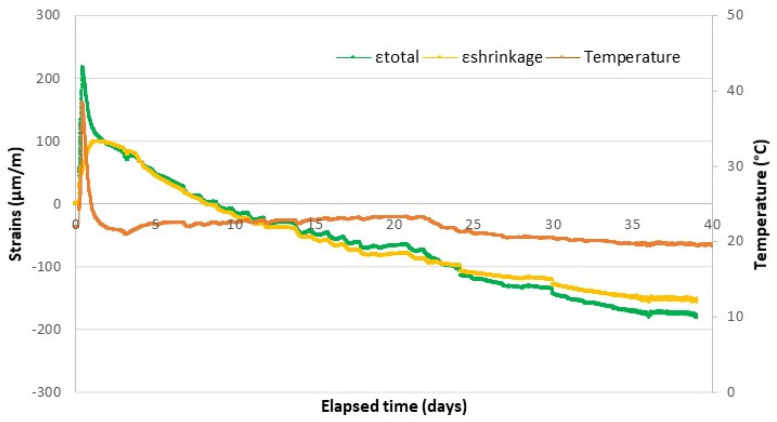
Strains and temperature with time. Prism under ambient conditions.

Figure 1 shows the total strains, the temperatures and the shrinkage strain measurements with the time for the concrete prism that remained at room temperature and environmental conditions.

As shown in Figure 1, the internal strains accumulate gradually to peak values, and then, the strains decrease slowly to finally end in stability after 35 days of measurements. It can be also observed from this figure that the total measured strain is an expansion strain and it experiences a rapid growth in the first 20 h of the test. From this point on, the total expansion strains begin to decrease, and from the ninth day of measurement onwards, it turns into a contraction (shrinkage) strain that keeps growing, at a small rate, until the end of the measurements. In fact, the evolution of micro-strain is strongly influenced by the cement hydration reactions as well as the temperature levels, as has already been reported by previous research [43]. The same behavior can be observed for the shrinkage strains. The results obtained indicate that the FBG sensors scheme used were efficient in capturing the expected behavior of early-shrinkage strains in concrete; an aspect that had already been reported by other researches [44,45].

**Figure 2:**
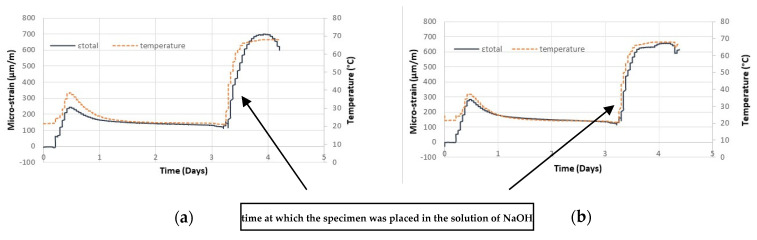
Temperature and strain measurements in: (**a**) prism 2 and (**b**) prism 3.

Figure 2 shows the total strains and the temperature levels observed in the two prisms that were placed into an aggressive environment in order to accelerate the internal expansion reactions. The strategy used to obtain this acceleration was described in the beginning of Section 3 of the paper.

**Figure 3:**
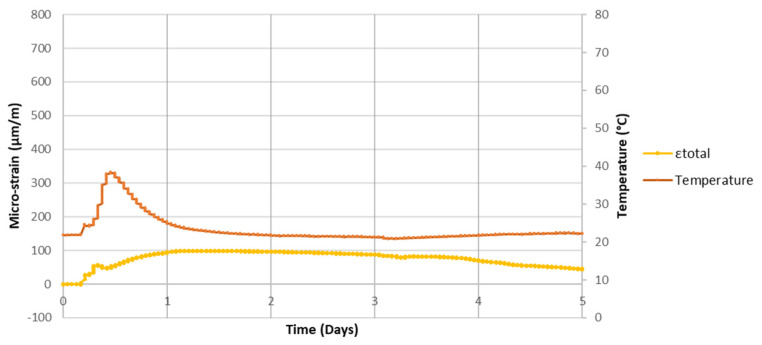
Strains and temperature with time. Prism under ambient conditions (detailed information of the first day of the test).

If one compares the behavior of the three prisms investigated—one placed at ambient conditions (Figure 3) and the two placed into a tank with the aggressive environment described (Figure 2)—it is possible to conclude that the sensors exhibited quite similar behaviors during the first 12 h of the test; temperature level close to 38 °C and strain level close to 250 μm/m.

At the time when the prims 2 and 3 were plane into the tank full of aggressive water, from the third day of the test, an accelerated and important increase in both total strains and temperatures was observed (total strain growth rate about 1500 μm/m/day). This was caused by the acceleration of expansive reactions, which was generated by the sodium hydroxide content added to the water used to make the concrete, and also to the water in which the prisms were immersed.

It is also important to highlight that the FOS and the scheme of the tests developed worked well in capturing the early strains in the concrete prisms investigated. The lack of strain measures after the fourth day of the test with the prisms placed inside the tank (Figure 2) was due the strong deterioration process that occurred in the FOS used, but the test scheme developed showed to be promising for measuring internal expansion in concrete, as the prism that was kept under ambient conditions the strain measurements proved to work well for 40 days. Research is underway to identify the best protection for fiber optic sensors so that they can be used for strain measurements over longer periods of time.

## 5. Conclusions

FOS technology has been successfully used for monitoring several types of concrete structures. The application of this type of technology has been increasingly frequent and promising. These are complex studies that need to be planned and performed in a conscious way for the results to be valid.

Most of the existing tests with FOS are carried out in a laboratory setting; however, there are already tests on real structures, mainly on bridges and viaducts, and some tests on buildings. The studies discussed in this paper highlight the potential for monitoring the performance of reinforced concrete structures with the use of FOS. On the other hand, there are important challenges to be overcome, especially the aspects related to data processing and the issues associated with the degradation of the sensors, mainly if one intends to use embedded FOS to assess expansions from AAR.

Moreover, the information about the equipment used and a justification for choosing the same ones is not always well-detailed in the existing works. This fact greatly limits the possibility of reproducing the same tests in a laboratory, or using this approach in real concrete works.

From the bibliographical review performed, one can say that there are currently several types of fiber optic sensors available on the technical market. These are usually sensors with multiple purposes, and they have been effective in several works investigated, especially in the study of deformations in concrete elements.

Internal expansion in concrete may come from different internal swelling reactions and the investigation of FOS to measure such expansion is an open field of research; few studies have been found on this topic, although its use is promising. Most studies were conducted to measure strains due, almost exclusively, to mechanical loads imposed on concrete specimens. In the specific case of internal expansions due to AAR, the measurement scheme with FOS proved to be functional and operational and it presents several advantages when compared with the current methods of expansion measurements, usually prescribed in AAR test code reading recommendations. Among these advantages, the research performed in this paper shows that it is possible to have an automatic and continuous availability of results of expansion data and, at the same time, assure improvements in the storage conditions and operations to read expansions from AAR in concrete samples.

## Figures and Tables

**Table 1:** Fiber Bragg Grating properties.

Parameter	Value
Young’s modulus [GPa]	70
Poisson ratio	0.17
Elastic-optical coefficients of silica (P_11_)	0.113
Elastic-optical coefficients of silica (P_12_)	0.252
FBG sensor length [mm]	5
Fiber-dependent constant, K_T_	0.0000078
Fiber-dependent constant, K_ε_	0.00000078

## Data Availability

The data that support the findings of this study are available upon request from the authors.
